# Histological heterogeneity in a large clinical cohort of juvenile idiopathic inflammatory myopathy: analysis by myositis autoantibody and pathological features

**DOI:** 10.1111/nan.12528

**Published:** 2019-03-11

**Authors:** S. A. Yasin, P. W. Schutz, C. T. Deakin, E. Sag, H. Varsani, S. Simou, L. R. Marshall, S. L. Tansley, N. J. McHugh, J. L. Holton, L. R. Wedderburn, T. S. Jacques, the UK Juvenile Dermatomyositis Research Group (UK and Ireland), Kate Armon, Joe Ellis‐Gage, Holly Roper, Vanja Briggs, Joanna Watts, Liza McCann, Ian Roberts, Eileen Baildam, Lou Hanna, Olivia Lloyd, Susan Wadeson, Phil Riley, Ann McGovern, Clive Ryder, Janis Scott, Beverley Thomas, Taunton Southwood, Eslam Al‐Abadi, Sue Wyatt, Gillian Jackson, Tania Amin, Mark Wood, Vanessa VanRooyen, Deborah Burton, Joyce Davidson, Janet Gardner‐Medwin, Neil Martin, Sue Ferguson, Liz Waxman, Michael Browne, Mark Friswell, Helen Foster, Alison Swift, Sharmila Jandial, Vicky Stevenson, Debbie Wade, Ethan Sen, Eve Smith, Lisa Qiao, Stuart Watson, Claire Duong, Helen Venning, Rangaraj Satyapal, Elizabeth Stretton, Mary Jordan, Ellen Mosley, Anna Frost, Lindsay Crate, Kishore Warrier, Stefanie ord, Clarissa Pilkington, Nathan Hasson, Sue Maillard, Elizabeth Halkon, Virginia Brown, Audrey Juggins, Sally Smith, Sian Lunt, Elli Enayat, Laura Kassoumeri, Laura Beard, Katie Arnold, Yvonne Glackin, Stephanie Simou, Beverley Almeida, Kiran Nistala, Raquel Marques, Stefanie Dowle, Charis Papadopoulou, Cer Johnson‐Moore, Emily Robinson, Kevin Murray, John Ioannou, Linda Suffield, Muthana Al‐Obaidi, Helen Lee, Sam Leach, Helen Smith, Anne‐Marie McMahon, Heather Chisem, Ruth Kingshott, Nick Wilkinson, Emma Inness, Eunice Kendall, David Mayers, Ruth Etherton, Danielle Miller, Kathryn Bailey, Jacqui Clinch, Natalie Fineman, Helen Pluess‐Hall, Lindsay Vallance, Lou Akeroyd, Alice Leahy, Amy Collier, Rebecca Cutts, Emma Macleod, Hans De Graaf, Brian Davidson, Sarah Hartfree, Danny Pratt

**Affiliations:** ^1^ Infection, Immunity, Inflammation Programme UCL GOS Institute of Child Health London UK; ^2^ Department of Pharmacy and Pharmacology University of Bath Bath UK; ^3^ Department of Molecular Neuroscience MRC Centre for Neuromuscular Diseases UCL Institute of Neurology London UK; ^4^ Arthritis Research UK Centre for Adolescent Rheumatology at UCL, UCLH and GOSH London UK; ^5^ NIHR Biomedical Research Centre at Great Ormond Street Hospital London UK; ^6^ Developmental Biology and Cancer Programme Developmental Biology of Birth Defects UCL GOS Institute of Child Health London UK; ^7^ Division of Neuropathology UCL Institute of Neurology London UK; ^8^ Department of Histopathology Great Ormond Street Hospital for Children NHS Foundation Trust London UK; ^9^ Present address: Department of Pathology and Laboratory Medicine University of British Columbia Vancouver BC Canada; ^10^ Present address: Division of Paediatric Rheumatology Department of Paediatrics Hacettepe University Faculty of Medicine Ankara Turkey

**Keywords:** anti‐SRP, dermatomyositis, immune mediated necrotizing myopathy, JDM score tool, polymyositis, principal component analysis

## Abstract

**Aim:**

Juvenile idiopathic inflammatory myopathies have been recently reclassified into clinico‐serological subgroups. Myopathological correlates of the subgroups are incompletely understood.

**Methods:**

We studied muscle biopsies from 101 children with clinically and serologically defined juvenile idiopathic inflammatory myopathies from the UK JDM Cohort and Biomarker Study by applying the international JDM score tool, myopathological review and C5b‐9 complement analysis.

**Results:**

Autoantibody data were available for 90/101 cases with 18/90 cases positive for anti‐TIF1γ, 15/90 anti‐NXP2, 11/90 anti‐MDA5, 5/90 anti‐Mi2 and 6/90 anti‐PmScl. JDM biopsy severity scores were consistently low in the anti‐MDA5 group, high in the anti‐Mi2 group, and widely distributed in the other groups. Biopsies were classified histologically as perifascicular atrophy (22/101), macrophage‐rich necrosis (6/101), scattered necrosis (2/101), clustered necrosis (2/101), inflammatory fibre invasion (2/101), chronic myopathic change (1/101), diffuse endomysial macrophage infiltrates (40/101) and minimal change (24/101). MDA5 cases segregated with the minimal change group and showed no capillary C5b‐9‐deposition. The Mi2 group displayed high severity scores and a tendency towards sarcolemmal complement deposition. NXP2 and TIF1γ groups showed a variety of pathologies with a high proportion of diffuse endomysial macrophage infiltrates and a high proportion of capillary C5b‐9 deposition.

**Conclusion:**

We have shown that juvenile idiopathic inflammatory myopathies have a spectrum of histopathological phenotypes and show distinct complement attack complex deposition patterns. Both correlate in some cases with the serological subtypes. Most cases do not show typical histological features associated with dermatomyositis (e.g. perifascicular atrophy). In contrast, more than half show relatively mild histopathological changes.

## Introduction

Juvenile idiopathic inflammatory myopathies (IIM) are a group of diseases comprising immunologically mediated inflammatory muscle diseases in children. This group of diseases is being re‐appraised and reclassified based on an improved understanding of the autoantibody landscape and clinical phenotypes. In the traditional clinico‐pathological classification, juvenile IIMs include juvenile dermatomyositis, polymyositis and cases that ‘overlap’ with other connective tissue diseases. A parallel serological classification based on myositis‐specific antibodies (MSA) and myositis‐associated antibodies (MAA) has emerged over the past decade. Initial correlation between the clinical findings and autoantibody groups revealed subphenotypes allowing a refined clinical description of these diseases 1. There are few studies analysing the correlation between the serological subtypes and muscle biopsy findings in adults and very few for juvenile IIM.

Early classification of IIMs on histopathological grounds into dermatomyositis, polymyositis, and inclusion body myositis was based on histochemical and morphological criteria. In 2003, an enlarged morphological ENMC classification also comprising nonspecific myositis and immune mediated necrotizing myopathies was published based on an international workshop consensus 2. The serological data in adult IIM patients suggested morphological and immunohistochemical subtypes for antisynthetase autoantibody associated myositis 3, 4, 5, MDA5‐associated dermatomyositis 6, and a correlation between HGMCR and SRP serological groups and immune mediated necrotizing myopathy 7. Differences in C5b‐9 deposition pattern have been suggested for anti‐Mi2 and anti‐TIF1γ serological groups 8, 9. Integration of these and other findings into a clinico‐sero‐pathological classification for adult IIMs is ongoing 9, 10, 11. In contrast, juvenile IIMs are rare and few sero‐pathological correlations have been reported. Our group has previously shown differences in histopathological severity between the MDA5 and Mi2 serological groups 12, 13 and described distinctive features of the paediatric patients positive for MDA5 13. SRP and HMGCR serological groups appear to be associated with necrotizing myopathies in children as they are in adults 14, 15, 16. Detailed histological studies in large serologically well‐defined cohorts have not been published to date, partly due to the rarity of such cohorts. The UK Juvenile Dermatomyositis cohort and biomarker study (JDCBS) and repository provides a unique opportunity in this regard.

Several rare and less well‐correlated myopathological manifestations of inflammatory muscle disease have been described in addition to the more frequent patterns, including inflammatory myopathy with abundant macrophages (IMAM) 17 and regional infarct‐like change 18. Furthermore, in clinical practice, muscle biopsies often have variable or nonspecific findings in children clinically suspected to have an immune‐mediated condition. These observations show a potential spectrum of histopathological findings seen in individual patients diagnosed with IIMs.

Our study describes systematically the biopsy pathology of a large cohort of clinically and serologically diagnosed juvenile IIM patients, and to test the hypothesis that specific features may be associated with autoantibody groups. This cohort allows an unbiased view of the pathological changes in these diseases, since patient selection was not based on histological criteria. We adopted a two‐stage approach. In the first stage, we analysed the biopsies in a standardized manner using our validated international JDM biopsy score tool 19, 20, and in a second stage we conducted an independent and blinded descriptive histopathological review of dominant pathological features.

## Methods

### Patients, biopsy material and clinical data

Paediatric patients with clinically diagnosed idiopathic inflammatory myopathy with or without skin changes were recruited to the UK JDCBS 21. Diagnosis was based on probable or definite Bohan and Peter Criteria. Written informed parental consent and age appropriate assent were obtained from participants prior to inclusion in the study. This research was approved by the Northern & Yorkshire Multi‐centre Research Ethics Committee, UK. Muscle biopsies from quadriceps were available for 101 patients in the JDCBS. Treatment status at time of biopsy, CK levels, clinical diagnosis and assessment were recorded. As a global measure of muscle strength, the Childhood Myositis Assessment Scale (CMAS; range 0–52 with high scores indicating normal strength) was applied in the majority of cases at the time of diagnosis.

### Stains and immunohistochemistry

Frozen sections were stained with Haematoxylin & Eosin, modified Gömöri trichrome (GT), NADH tetrazolium reductase and Acid Phosphatase. Immunohistochemical studies were performed as described previously 12, 19, 20. The following primary antibodies were used: anti‐human CD3 (UCHT1), anti‐human CD68 (KP1), anti‐human major histocompatibility complex class I heavy chain (W6/32) and anti‐human neonatal myosin (WB‐MHCn) from Novacastra, UK; anti‐human CD31 (JC70A) from Dako, Cambridgeshire, UK, and anti‐human C5b‐9 (Ae11) from Dako.

### Severity scoring of biopsies

Scoring of biopsy samples was conducted using the validated JDM biopsy score tool as described previously 19, 20. Briefly, severity of pathology was assessed in four domains: inflammatory, vascular, muscle fibre and connective tissue. The number of inflammatory cells infiltrating endomysial, perimysial and perivascular regions on CD3 and CD68 staining, was assessed and scored according to cell density and presence of clusters, as specified previously. Components of the muscle fibre domain used to determine abnormalities were over‐expression of MHC class I, expression of neonatal myosin, assessment of the number of atrophic, necrotic, degenerating and regenerating fibres within fascicles and in peri‐fascicular regions and presence of internal nuclei. Vascular pathology was assessed by determining loss of CD31 capillary staining, vessel abnormalities and infiltration and the presence or absence of infarction. Excess collagen and fibrosis was assessed on GT stains in endomysial and perimysial regions. The total score was calculated as the sum of the domain scores. Total biopsy scores across these domains can range from 0 to 27, with higher scores indicating more severe pathology. All biopsy severity scores were assessed by a single observer (SAY), who was blinded to the autoantibody status of each patient.

### Histological review of biopsies

In parallel with the use of the score tool, histological review of all 101 biopsies was carried out by a senior neuropathologist (PWS) independently of, and blinded to, scoring results, autoantibody status and clinical data. Stains and immunohistochemical studies were available as stated above. The aim of the histological review was a descriptive assessment of the dominant pathological features in each muscle biopsy. Groups were largely based on findings described in the context of typical myopathological diagnoses, such as perifascicular atrophy (PFA) in classic dermatomyositis, macrophage rich necrosis in IMAM 17, inflammatory fibre invasion (FI) in inclusion body myositis, scattered necrosis (SN) in immune mediated necrotizing myopathies, and clustered necrosis (CN) in regional ischemic immune myopathy 18. It is important to emphasize that we wish to distinguish between descriptive histological groups used for this study and the diagnostic entities related to them.

### Autoantibody screening

Serum or plasma were screened for autoantibodies using immunoprecipitation as described previously 12. The following controls were used as standard: Normal serum, anti‐Jo1, ‐PL7, ‐PL12, ‐Zo, ‐U1, ‐RNAPII, ‐PmScl, ‐Ro60, ‐La, ‐Mi2, ‐Ku, ‐SAE, ‐RNAPI/III, ‐U3, ‐TIF1γ, ‐SRP, ‐Scl70 and 140 kDa band. ELISA was used to confirm specificity for anti‐NXP2 or anti‐MDA5 in patients with a 140 kDa band. All specimens were additionally screened by ELISA for anti‐HMGCR antibody. If no autoantibody bands at all were detected, the case was put in the ‘NIL’ group. It is important, however, to note that the absence of detectable bands on immunoprecipitation does not exclude the presence of autoantibodies. For some analyses, autoantibody bands of unknown specificity were present. The group of ‘no identifiable autoantibodies’ (NIA) comprised those cases with no bands and those with an unidentifiable band. For initial evaluations of score tool data, the NIL group was used as comparison group. For analysis of the histopathological categories, the NIA group was felt to be more comprehensive and better suited.

### Statistical analysis

Principal component analysis (PCA) was performed on the entire cohort to reduce the number of dimensions in the severity score data and enable visualization of potential underlying clusters. PCA analysis was performed using the R package FactoMineR 22. Vascular domain features were excluded because they displayed the least variance. Steroid treatment was included as parameter to control for its effects. Visualization of the PCA analysis used the coordinates for each patient on the first two principal components, coloured according to the autoantibody group.

The distributions of domain score totals, CMAS score and dominant fibre pathology categories across autoantibody groups were analysed by Kruskal–Wallis test (GraphPad Prism 5 GraphPad Software, La Jolla, California, USA). For categorical variables, groups with small sample size (*n* < 2) were excluded from statistical analysis. *Post‐hoc* comparisons were performed using Dunn's Multiple Comparison Test. Differences in autoantibody frequency across histological groups were analysed by the chi square test. For all analyses, *P*‐values of <0.05 were considered statistically significant. PCA of score tool data and subsequent analysis of domain specific data is based on all 101 cases, including cases treated with steroids at the time of biopsy, since steroid treated did not show a significant effect on total severity scores, as shown by Mann–Whitney test on total severity scores, *P *= 0.52. All cases were individually reviewed and assigned to histological groups. Analysis of the correlation of histological groups with serological and clinical data was restricted to cases without treatment at the time of biopsy.

## Results

One hundred and one biopsies from patients with juvenile idiopathic inflammatory myopathy were included in this study. Table 1 shows the demographic and clinical features of the group including autoantibody results. Antibodies against Jo1, PL12, Zo, Ro60, La, Ku, RNAPII, RNAPI/II, U3, Scl70 and HMGCR were not detected in this cohort.

**Table 1:** Demographic, clinical and serological features (*n* = 101)

Feature	Summary statistic
Gender, *n* (%) M:F	33 (32.7):68 (67.3)
Ethnicity, *n* (%)	
White	72 (71.3)
Black	12 (11.9)
South Asian	8 (7.9)
Other	9 (8.9)
Clinical features, median [interquartile range]	
Age at onset (years)	6.1 [3.9–9.3]
Time from disease onset to diagnosis, (months)	2.6 [1.5–7.5]
Time from diagnosis to biopsy, (months)	0.72 [0.39–0.92]
On steroids at biopsy, *n* (%)	12 (12.2)
CMAS at biopsy	29 [18.75–45]
Reported clinical diagnosis, *n* (%)	
Definite or probable juvenile dermatomyositis	88 (88)
Juvenile dermatomyositis overlap with scleroderma	2 (2)
Juvenile dermatomyositis overlap with polyarthritis	6 (6)
Definite or probable Juvenile polymyositis	2 (2)
Focal myositis	1 (1)
Mixed connective tissue disease	1 (1)
Other idiopathic inflammatory myopathy	1 (1)
Antibody Status, *n*	90 of 101
Myositis‐specific autoantibodies, *n* (%)	53 of 90 (58.9)
Anti‐TIF1γ	18 (20.0)
Anti‐NXP‐2	15 (16.7)
Anti‐MDA5	11 (12.2)
Anti‐Mi2	5 (5.6)
Anti‐SRP	2 (2.2)
Anti‐PL7	1 (1.1)
Anti‐SAE	1 (1.1)
Myositis‐associated autoantibodies, *n* (%)	9 of 90 (10.0)
Anti‐PM‐Scl	6 (6.7)
Anti‐U1RNP	2 (2.2)
Anti‐Scl70	1 (1.1)
No identifiable autoantibodies, *n* (%)	28 of 90 (31.1)

Autoantibody status data were available in 90 cases. Percentages reflect the number of patients with a given antibody as a proportion of total tested patients.

CMAS, Childhood Myositis Assessment Scale.

### Pathological severity scores were distributed widely from mild to severe across the entire cohort

All 101 muscle biopsies in the cohort were assessed using the validated JDM muscle biopsy score tool 19, 20. This tool measures the severity of pathology in muscle biopsies in four domains (inflammatory, muscle fibre, vascular, connective tissue). Total scores across the entire cohort had a wide distribution with a range from 2 (almost normal) to 26 (severe); an interquartile range of 10–21; and a median of 17. We hypothesized that this variability is related to the differing underlying autoantibody groups in the cohort. To test the possibility that variability was related to serological type, we analysed the data according to autoantibody subgroups.

### Autoantibody stratification showed differences in total severity scores between MDA5 and Mi2 groups

**Figure 1:**
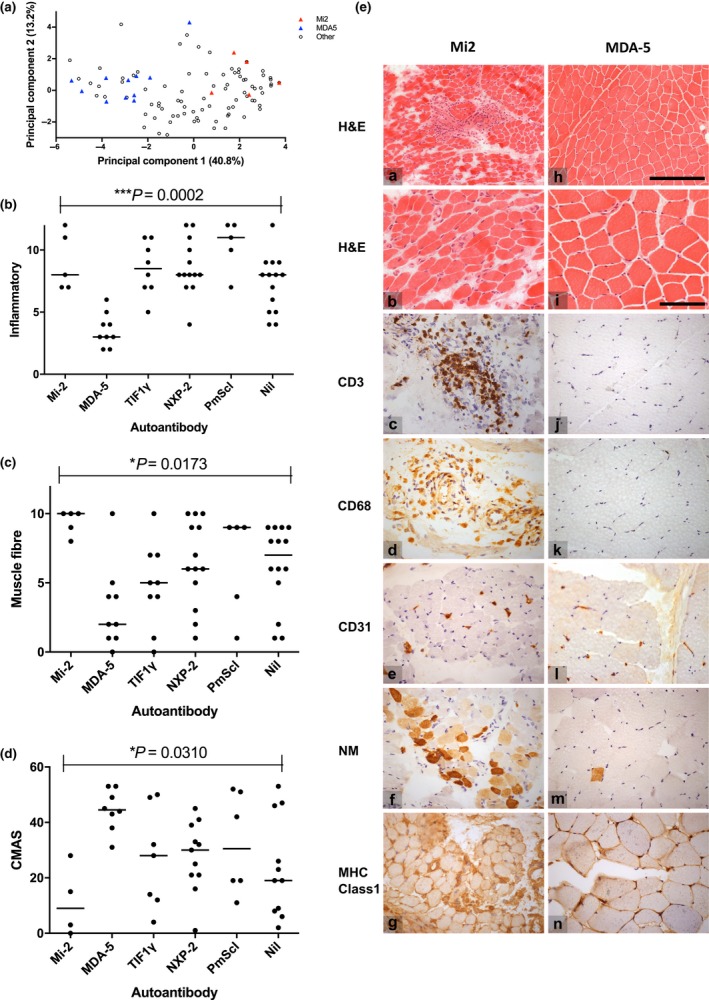
MDA5 and Mi‐2 autoantibody groups define clusters of mild and severe pathology (**a**) Principal component analysis of each feature of the biopsy tool for each biopsy sample. Each symbol indicates an individual case, plotted on the two principal components that capture the most variance in the data, with the percentage of variance captured given in parentheses. Triangles are coloured according to auto‐antibody groups as indicated. (**b**,**c**) Domain totals for each of the domains in the juvenile dermatomyositis biopsy score tool according to autoantibody subgroups. The inflammatory domain represents the sum of scores for CD3 and CD68 positive endomysial, perimysial and perivascular cells, the muscle fibre domain represents the sum of scores for MHC class I expression, neonatal myosin expression, perifascicular and non‐perifascicular atrophy and fibre degeneration and regeneration, and internal nucleation. Kruskal–Wallis anova was performed to compare the distribution of domain totals across myositis‐specific antibodies/myositis‐associated antibodies subgroups. *Post‐hoc* analysis indicated that the inflammatory domain scores differed between the anti‐Mi2 and anti‐MDA5, anti‐MDA5 and anti‐TIF1γ, and anti‐MDA5 and anti‐NXP2 cases. The muscle fibre domain scores differed between the anti‐Mi2 and anti‐MDA5 cases. Nil: no autoantibody bands detected on immunoprecipitation analyis. (**d**) Correlation of autoantibody group and muscle weakness, as assessed by the Childhood Myositis Assessment Scale (CMAS). Kruskal–Wallis anova was used to compare the degree of muscle weakness across autoantibody subgroups. *Post‐hoc* comparisons indicated that CMAS differed between the anti‐Mi2 and anti‐MDA5 cases, with Mi2 cases being much weaker. (**e**) Representative micrograph images of representative Mi2 (a–g) and MDA5 (h–n) muscle biopsies. Mi2 cases typically had high levels of inflammation, fibre damage and vessel abnormalities, while MDA5 cases had a much more normal appearance with only mild fibre size variation. a,b,h,i – Haematoxylin & Eosin (H&E), c,j – CD3, d,k – CD68, e,l – CD31, f,m – neonatal myosin, g,n – MHC class1. Scale bars for micrographs a and h are shown in h, length 250 μm, scale bars for b–g and i–n shown in i, length 100 μm.

As we have previously shown, muscle biopsy scores for patients with MDA5‐associated myopathies cluster in the mild range, Mi2‐associated cases cluster in the severe range, and TIF1γ‐ and NXP2‐associated cases vary across a wide range, Figure 1 12. To further analyse these findings, we conducted PCA using all 101 sets of biopsy score data. The first principal component, which captured 40.8% of the total variance in the pathological scores, separated the anti‐MDA5 and anti‐Mi2 cases into separate clusters (Figure 1 **a**). No other myositis specific autoantibody subgroups separated into clusters when projected onto the first two principal components, indicating that the score tool data could not explain the underlying variability in these groups. To better understand the source of this variability of the total scores, we next investigated the domain specific score data.

### Differences in total scores were accounted for by variability in the myofibre and inflammatory subdomains

In the PCA, inflammatory and muscle fibre domain scores accounted for a large proportion of the variance between these two groups. Domain specific analysis of the score tool data confirmed that the severity of inflammatory infiltrates and of fibre pathology differ between Mi2 and MDA5 antibody subgroups, whereas other myositis specific autoantibody groups, in particular TIF1γ and NXP2, had a wide range of scores for severity in these domains. (Figure 1 **b**,**c**). No significant differences between groups were seen in the vascular and connective tissue domains (data not shown). Severity scores correlated weakly with muscle strength at time of biopsy as we have shown previously 19, and MSA status also showed associations with muscle strength at the time of biopsy (Figure 1 **d**).

Preliminary histological examination showed that all patients with autoantibodies to Mi‐2 had significant pathological change in their biopsies (Figure 1 **e**). In particular, there were high levels of inflammation and a range of fibre pathology including PFA, necrosis and severe chronic myopathic changes. In contrast, the majority of patients with autoantibodies to MDA5 had very mild, almost normal, muscle biopsies, with little or no inflammation and minimal myofibre abnormalities, such as increased fibre size variability (Figure 1 **e**). However, eight of nine cases showed increased MHC Class I expression.

Representative biopsy pathology for a patient with Mi2 and MDA5 autoantibodies are shown in Figure 1 **e**, with intense inflammatory infiltrates and muscle fibre damage in the Mi2 group and minimal changes (MC) in the MDA5 group.

The MDA5 group contained one outlier case both on PCA and domain‐specific analysis that did not cluster with other cases in the MDA5 group. This case scored higher for muscle fibre pathology, which showed PFA and SN (Figure 1 **b**). This patient also had a long time interval between disease onset and muscle biopsy of approximately 5 years, which could be related to the increased severity of pathology seen in this biopsy.

Overall, score tool data analysis demonstrated that severity of muscle pathology is not randomly distributed across autoantibody groups in juvenile IIM.

The score tool gives an overall measure of severity but does not indicate the underlying type of pathology. We hypothesized that there may be variation in the patterns of pathology that might explain the variability in the biopsies. Therefore, in our second analysis, biopsy histology was reviewed by an experienced pathologist who was blinded to score tool data, as well as the clinical and serological features. The primary aim was to determine if a pattern of pathology could be recognized in each biopsy and if so whether these findings correlated with serological or clinical data.

### JIIM patients show a variety of myopathological changes that could be summarized in distinct histopathological groups

**Figure 2:**
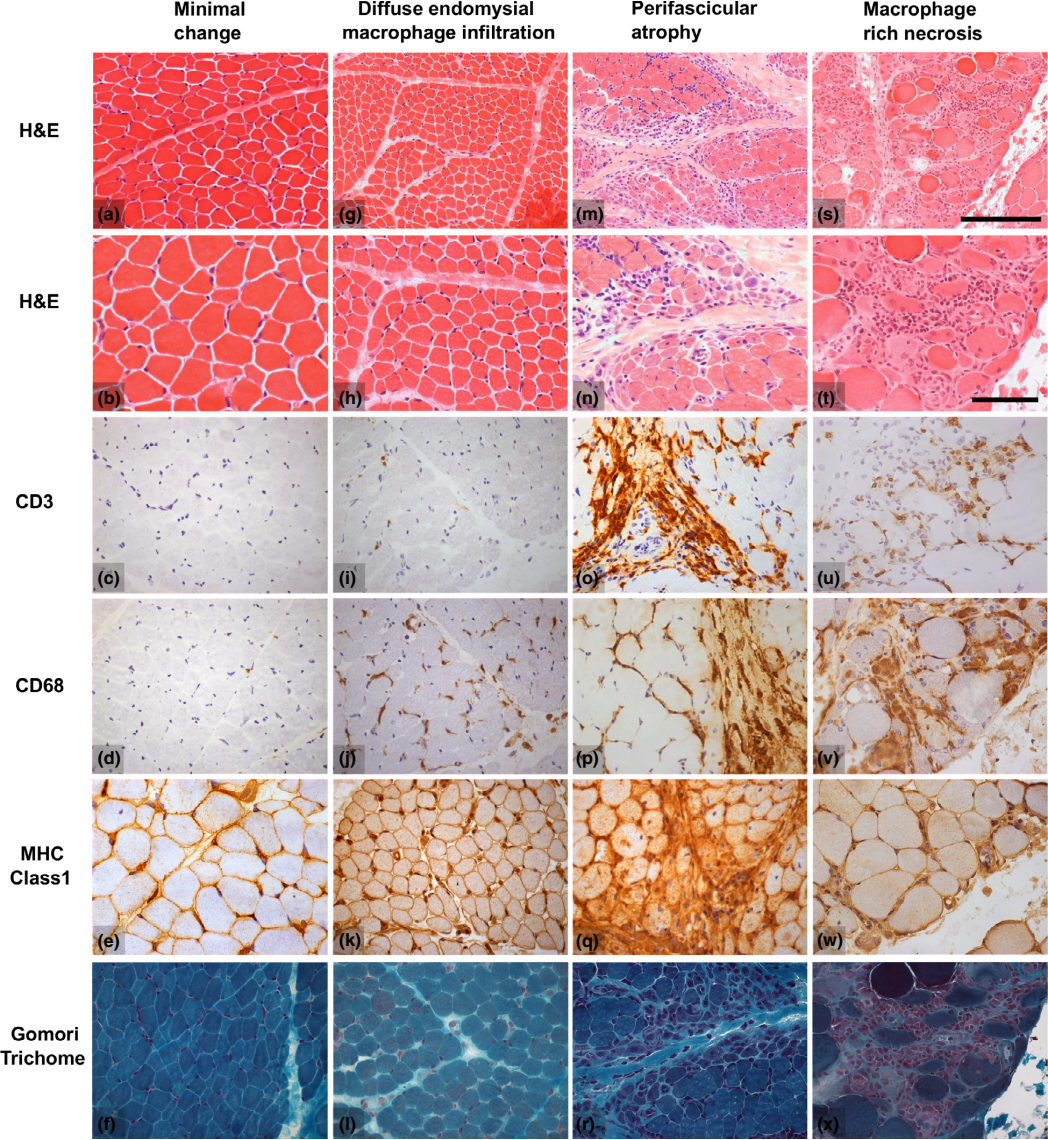
Illustrations of the histological features of the frequent histological groups in the biopsy cohort. The example of minimal change (**a**–**f**) demonstrates increased fibre size variability in the absence of macrophage infiltrates on CD68 immunohistochemistry. The example of diffuse endomysial macrophage infiltrates (**g**–**l**) also shows mild fibre pathology, but has increased endomysial CD68 labelling combined with overexpressed MHC class I. The illustration of perifascicular atrophy (**m**–**r**) shows several layers of atrophic cells in perifascicular distribution with marked peri‐ and endomysial lymphocytic infiltrates (CD3). Note that endomysial CD68 labelling is also increased and that necrosis is largely absent. The example of macrophage rich necrosis (**s**–**x**) shows numerous, often intrafascicular, necrotic fibres in various stages of myophagocytosis, a dense macrophage infiltrate (CD68) and a less dense lymphocytic infiltrate (CD3). Images chosen show typical changes although do not capture the full histological variability in each group. Scalebars: for micrographs **a**,**g**,**m**,**s**, bar shown in **s**, length 250 μm; for remaining micrographs bar shown in **t**, length 100 μm.

Descriptive categories and their frequency in this cohort are summarized in Table 2. There were several biopsies in our cohort with only mild abnormalities and no significant inflammatory infiltrates. We called this group of cases ‘MC’ (24/101 cases; 19/89 untreated cases; Figure 2 **a**–**e**). Next, we identified a group that was distinguished by diffusely increased endomysial CD68‐positive cells in combination with increased sarcolemmal MHC class I expression, which we called the ‘diffuse endomysial macrophage infiltration’ pattern (DEMI; 40/101 cases; 36/89 untreated cases; Figure 2 **f**–**j**). This group was formally defined by the presence of an endomysial macrophage infiltrate score of 2, combined with MHC class I overexpression, but no or only rare atrophic or necrotic muscle fibres. Biopsies with DEMI infiltrates occasionally displayed rare regenerating fibres or rare lymphocytic infiltrates, but by definition, these were not prominent features. Another large group was characterized by PFA (22/101 cases; 19/89 untreated cases; Figure 2 **k**–**o**). PFA affected more than one layer of atrophic fibres on the edge of at least one fascicle. Small numbers of necrotic fibres were sometimes observed, but these were not a dominant feature. Biopsies with PFA often had increased perimysial lymphocytic infiltrates, and endomysial and perimysial fibrosis. A small group of biopsies was characterized by extensive and diffuse fibre necrosis, numerous macrophages and moderate, often endomysial, lymphocytic infiltrates, which we designated as the ‘macrophage‐rich necrosis’ pattern (MRN; 6/101 cases; 6/89 untreated; Figure 2 **p**–**t**). Necrotic fibres in various stages of myophagocytosis were found in both peri‐ and intrafascicular location, scattered or confluent. PFA was sometimes present as a minor component.

**Table 2:** Frequency of histological groups in the biopsy cohort, *n* = 101

Dominant histological pattern	*n* (%)
Minimal change	24 (24)
Diffuse endomysial macrophage infiltrates	40 (40)
Perifascicular atrophy	22 (22)
Macrophage rich necrosis	6 (6)
Clustered necrosis	2 (2)
Scattered necrosis	2 (2)
Fibre invasion	2 (2)
Chronic myopathic	1 (1)
Other	2 (2)

**Figure 3:**
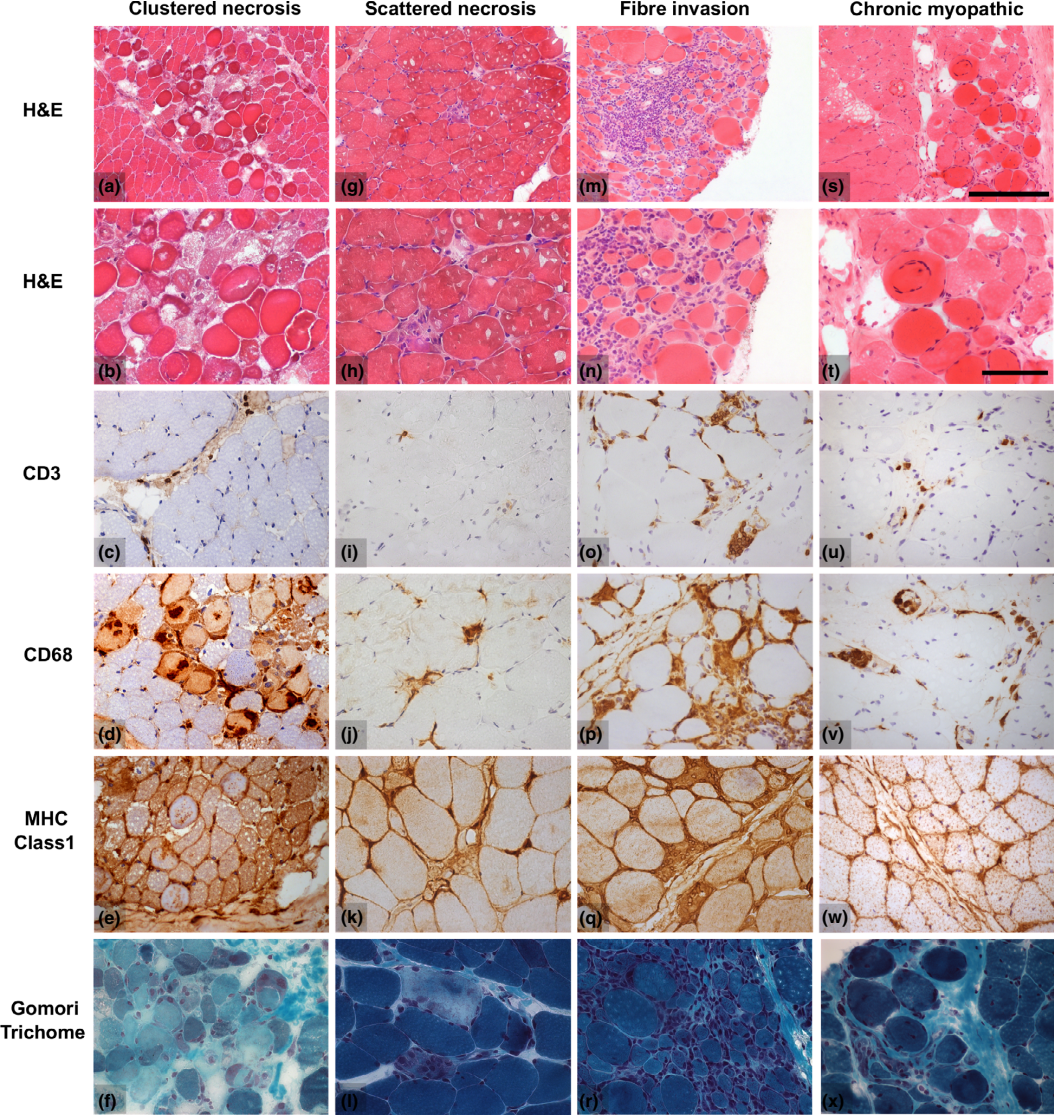
Illustrations of histological features of rare histological groups in the biopsy cohort. The example of clustered necrosis (**a**–**f**) illustrates an area of necrotic fibres surrounded by relatively normal appearing muscle in the absence of significant lymphocyte (CD3) or macrophage (CD68) infiltrates. Only occasional macrophages are present in necrotic fibres. Scattered necrosis (**g**–**l**) is reminiscent of the findings in immune‐mediated necrotizing myopathy. The example shows two scattered necrotic fibres undergoing myophagocytosis with macrophage infiltrates (CD68) and remarkably little inflammation (CD3). Two cases demonstrated inflammatory invasion of normal appearing muscle fibres in combination with endomysial lymphocytic infiltrates (**m**–**r**). These biopsies typically also had a significant number of necrotic fibres. A single biopsy showed only marked chronic myopathic change (**s**–**x**) with marked fibre size variability and hypertrophy, internal nucleation, whorled fibres and endomysial fibrosis. Images chosen show typical features, although they do not capture the full histological variability in each group. Scalebars: for micrographs **a**,**g**,**m**,**s**, bar shown in **s**, length 250 μm; for remaining micrographs, bar shown in **t**, length 100 μm.

Rare cases showed other distinctive pathologies. Two cases demonstrated clusters of necrotic fibres resembling a small infarct, termed ‘CN’, but no significant inflammatory infiltrates (2/101 cases; 2/89 untreated cases; Figure 3 **a**–**e**). Two cases had scattered necrotic fibres (2/101 cases; 2/89 untreated cases; Figure 3 **f**–**j**) with very little inflammation, as is typically described in immune‐mediated necrotizing myopathy. Two cases showed invasion of normal appearing muscle fibres by lymphocytes, grouped as inflammatory FI (2/101 cases; 2/89 untreated cases; Figure 3 **k**–**o**). A single biopsy exclusively showed features of a severe chronic myopathy (Figure 3 **p**–**t**) with marked fibre size variation, internal nucleation and frequent whorled fibres. Finally, two biopsies could not be assigned to any of the above groups and were classified in an ‘other’ category. One of these showed features of neurogenic change and the other of diffuse fibre necrosis at a similar stage of phagocytosis consistent with rhabdomyolysis (the clinical impression in this case was described as ‘focal myositis’).

### Rare biopsies showed combinations of main pathological features

The majority of biopsies displayed features which fell into one of the categories defined above. In just four cases were two distinct patterns seen in different areas of the biopsy. Two cases had PFA and macrophage rich necrosis. One of these was from a patient with anti‐Mi2 antibody, the other from the patient with anti‐MDA5 antibody who had a long disease course before biopsy, as mentioned above. These cases were excluded from the further correlational analysis. Another two cases showed marked chronic myopathic change in addition to PFA. These cases were classified as PFA with the chronic myopathic change assumed to be secondary.

### Biopsies with DEMI infiltrates have a serological profile which is distinct from MC biopsies

**Figure 4:**
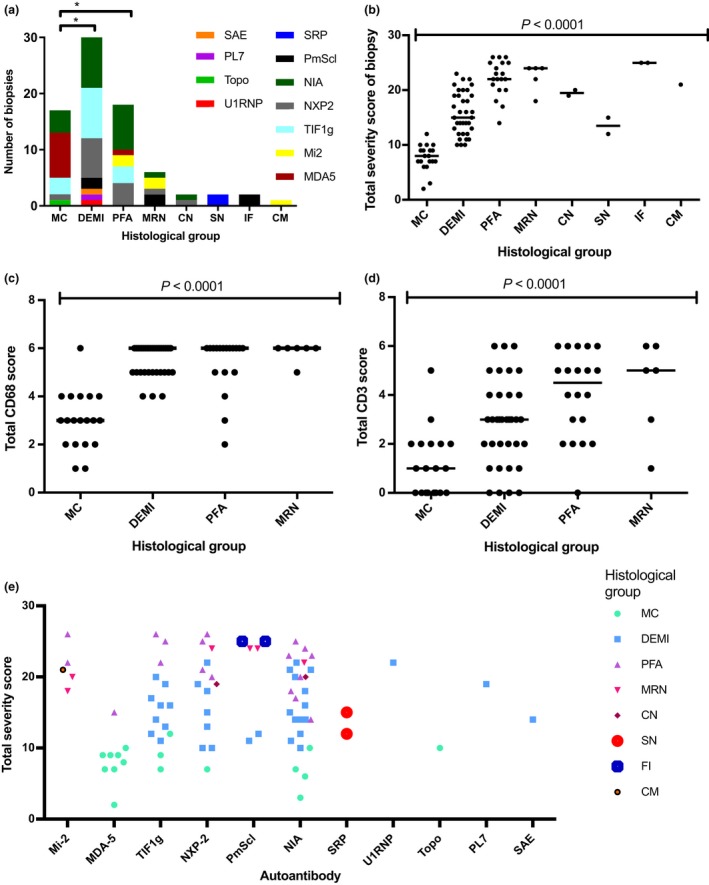
Serological profiles of the histological groups and correlation of the histological groups with the JDM severity score tool results. Patients were included in the analysis if they were untreated at the time of biopsy and if serological data were available (83/101). (**a**) Analysis of serological profiles of the histological groups. The MC group has a significantly different serological profile from the DEMI group (*P* = 0.002) and the PFA group (*P* = 0.041; Chi‐square test), while no statistically significant difference was apparent between DEMI and PFA (*P* = 0.3) or between PFA and MRN (*P* = 0.08); chi‐square test for all comparisons; *P < 0.05. (**b**) Total severity score from the international score tool as correlated with dominant fibre pathology. (**c**,**d**) Correlation of more frequent pathological patterns with lymphocytic and macrophage infiltrates. Total CD3 and CD68 scores are the sums of endomysial, perimysial and perivascular scores from the international score tool. (**e**) Plot of severity scores across autoantibody groups subclassified according to dominant fibre pathology. MC, minimal change; DEMI, diffuse endomysial macrophage infiltrates; PFA, perifascicular atrophy; MRN, macrophage rich necrosis; CN, clustered necrosis; SN, scattered necrosis; FI, fibre invasion; CM, chronic myopathic change.

To test the significance of the histopathological differences between MC and DEMI (DEMI) cases, we compared the autoantibody profile of both groups restricting biopsies to patients without steroid treatment at the time of biopsy. Analysis of the major myositis‐specific autoantibody groups demonstrated a clearly distinct distribution (Figure 4 **a**): MC cases account for almost all of the MDA5 group whereas DEMI cases were mostly NXP2 or TIF1γ, or NIA cases. None of the MC or DEMI cases had Mi2 antibodies. These results suggest that the distinction between MC and DEMI may be biologically significant. Differences between these two groups were apparent upon analysis of associated inflammatory infiltrates with generally higher lymphocyte scores in DEMI compared to MC (Figure 4 **b**,**c**). Histological groups correlated well with distinct ranges of JDM severity scores (Figure 4 **d**).

### NXP2, TIF1γ and Mi2 groups were myopathologically heterogeneous compared to the MDA5 group, which was characterized by MC

The myositis‐specific autoantibody groups NXP2, TIF1γ and Mi2 showed more than one pathological pattern on biopsy (Figure 4 **e**). In the NXP2 and TIF1γ serological groups, a combination of biopsies with DEMI infiltrates and PFA was present, explaining partly the variability of the severity scores observed in the first stage of this study. Interestingly, there were also other pathological patterns present, illustrated by a case with CN and one case with macrophage rich necrosis in the NXP2 (Figure 4 **e**). The Mi‐2 group appeared more homogeneous on score tool analysis with generally high severity scores, but upon histological review also showed heterogeneous histological patterns with two cases of PFA, one case of macrophage rich necrosis and one case with chronic myopathic change (Figure 4e). All biopsies from untreated patients in the MDA5 group showed MC pathology with notable absence of inflammatory infiltrates and very little fibre pathology, with one exception noted above.

### Complement attack complex deposition patterns vary between serological subgroups

**Figure 5:**
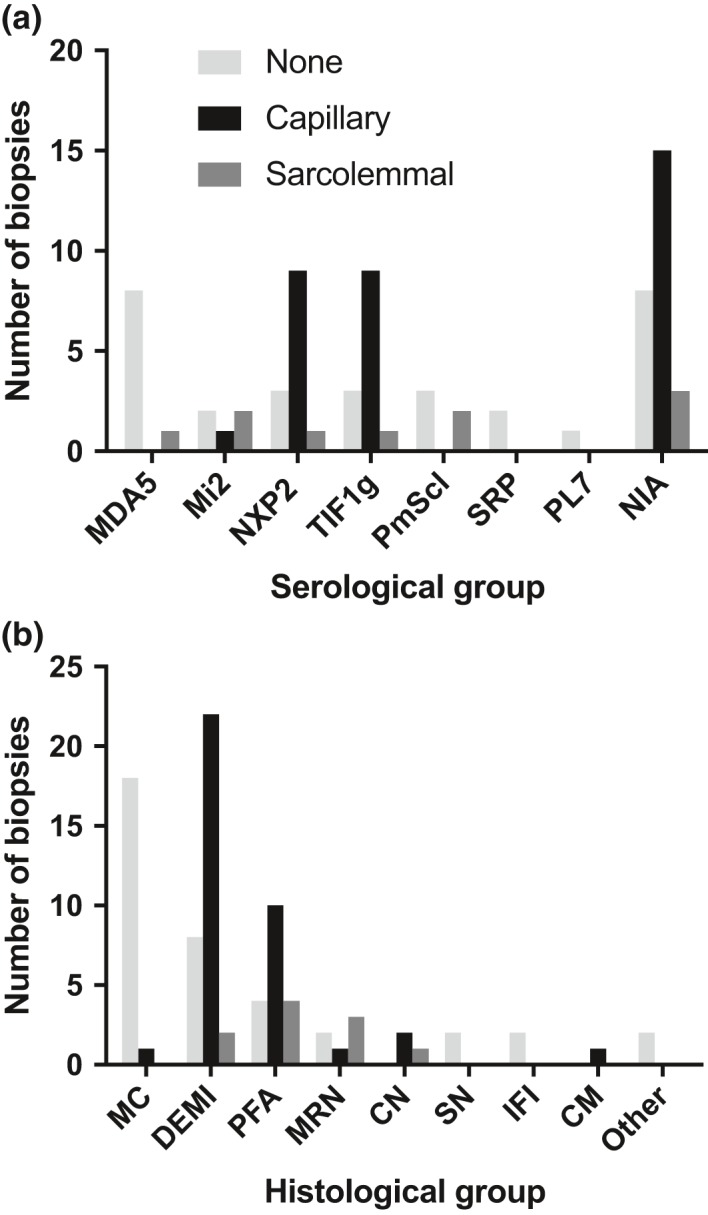
Correlation of complement attack complex (C5b‐9) deposition pattern with serological and histological groups across the study cohort. The results of immunohistochemical staining for C5b‐9 were available for 76 patients after restriction to patients not on steroid treatment at the time of biopsy. Patient numbers whose biopsies showed capillary complement deposition, sarcolemmal complement deposition, or neither are plotted against (**a**) serological and (**b**) histological groups. For statistical analysis cases with sarcolemmal membrane attack complex deposition and without any deposition were merged into a single category in order to ensure meaningful group sizes. For results see text. MC, minimal change; DEMI, diffuse endomysial macrophage infiltrates; PFA, perifascicular atrophy; MRN, macrophage rich necrosis; CN, clustered necrosis; SN, scattered necrosis; FI, fibre invasion; CM, chronic myopathic change. NIA, no identifiable autoantibodies.

C5b‐9 immunohistochemical studies were available for 96/101 biopsies and for 76/89 biopsies from untreated patients. Figure 5 shows the results of complement labelling in a capillary or sarcolemmal pattern or no labelling, defined as neither capillary, nor sarcolemmal, across serological and histological groups for untreated cases. Capillary deposition pattern consisted of at least 2–3 clustered and dot‐like complement deposits in capillaries, whereas sarcolemmal deposition consisted of granular circumferential decoration of muscle fibre membranes. The negative groups showed neither pattern but may show complement deposition in necrotic fibres. The vast majority of cases with capillary C5b‐9 labelling were found in the TIF1γ and NXP2 groups, or the group with no identifiable antibodies. The Mi2 cases showed a increased proportion of sarcolemmal labelling compared to capillary labelling and the MDA5 group did not show cases with capillary labelling. For statistical analysis, we merged the groups with sarcolemmal complement deposition and with no complement deposition into a single category of no capillary complement deposition to ensure meaningful group sizes. Chi square analysis of the larger MSA groups (MDA5, Mi2, NXP2, TIF1γ) with variables grouped into capillary complement deposition and no capillary complement shows statistically differences between groups (*P* = 0.002). However, Fisher's exact test did not demonstrate differences between the NXP2 and Mi2 groups. Given the small numbers, however, a difference cannot be excluded. Labelling patterns analysed according to histological subgroup are in keeping with the serological profile of each group, considering that the main serological groups in DEMI and PFA are NXP2, TIF1γ and NIA (Figure 4 **a**). A summary of the correlation of serological groups with histological groups, complement deposition pattern and JDM severity scores in absolute numbers is presented in Table 3.

**Table 3:** Correlation of serological groups with JDM severity score, histological groups and complement deposition pattern

	Severity score	MC	DEMI	PFA	MRN	CN	SN	CM	IF	Other	Total	C5b‐9 cap	C5b‐9 sarc	C5b‐9 neg
MDA5	9 (7–9.5)	8	0	1	0	0	0	0	0	0	9	0/9	1/9	8/9
Mi2	21 (19–24)	0	0	2	2	0	0	1	0	0	5	1/5	2/5	3/5
TIF1γ	16 (12–20)	3	9	3	0	0	0	0	0	0	15	9/13	1/13	3/13
NXP2	19 (12–22.5)	1	7	4	1	1	0	0	0	0	14	9/13	1/13	3/13
SRP	13.5 (12–15)	0	0	0	0	0	2	0	0	0	2	0/2	0/2	2/2
PL7	19	0	1	0	0	0	0	0	0	0	1	1/1	0/1	1/0
SAE	14	0	1	0	0	0	0	0	0	0	1	1/1	0/1	1/0
PmScl	24 (12–25)	0	2	0	2	0	0	0	2	0	6	0/5	2/5	3/5
U1RNP	20 (18–22)	0	1	0	0	0	0	0	0	1	2	1/2	0/2	1/2
Scl70	10	1	0	0	0	0	0	0	0	0	1	1/1	0/1	1/0
NIA	17 (12–21)	4	13	8	1	1	0	0	0	0	27	15/24	3/24	6/24
Total		17	34	18	6	2	2	1	2	1	83			

Severity score is displayed as median (interquartile range). Other columns show patient numbers. Included are all patients who had serological analysis and who were without steroid treatment at the time of biopsy (83/101). C5b‐9 studies were not available for all patients and total numbers are indicated as fractions of total studies available in respective columns.

NIA, no identifiable autoantibody; MC, minimal change; DEMI, diffuse endomysial macrophage infiltrates; PFA, perifascicular atrophy; MRN, macrophage rich necrosis; CN, clustered necrosis; SN, scattered necrosis; IFI, inflammatory fibre invasion; CM, chronic myopathic; C5b‐9 cap, C5b‐9 deposition in a capillary pattern; C5b‐9 sarc, C5b‐9 deposition in a sarcolemmal pattern; C5b‐9 neg, neither sarcolemmal nor capillary complement deposition.

### Biopsy pathology in the PmScl and SRP autoantibody groups is in keeping with reports from adult patients

Several observations are warranted for the smaller autoantibody groups and for MAA. PmScl patients did not show PFA on biopsy, but a mixture of MRN, inflammatory FI and endomysial macrophage infiltrates. Conversely, all cases with inflammatory FI fell into the PmScl group in keeping with a traditionally assumed association of PmScl with polymyositis 23. There were two SRP cases in this cohort and both presented with a histological pattern of immune mediated necrotizing myopathy, in keeping with observations from adult cohorts. These cases have been independently reported 14.

### Clinical diagnosis and CMAS score do not correlate with descriptive histological groups

**Figure 6:**
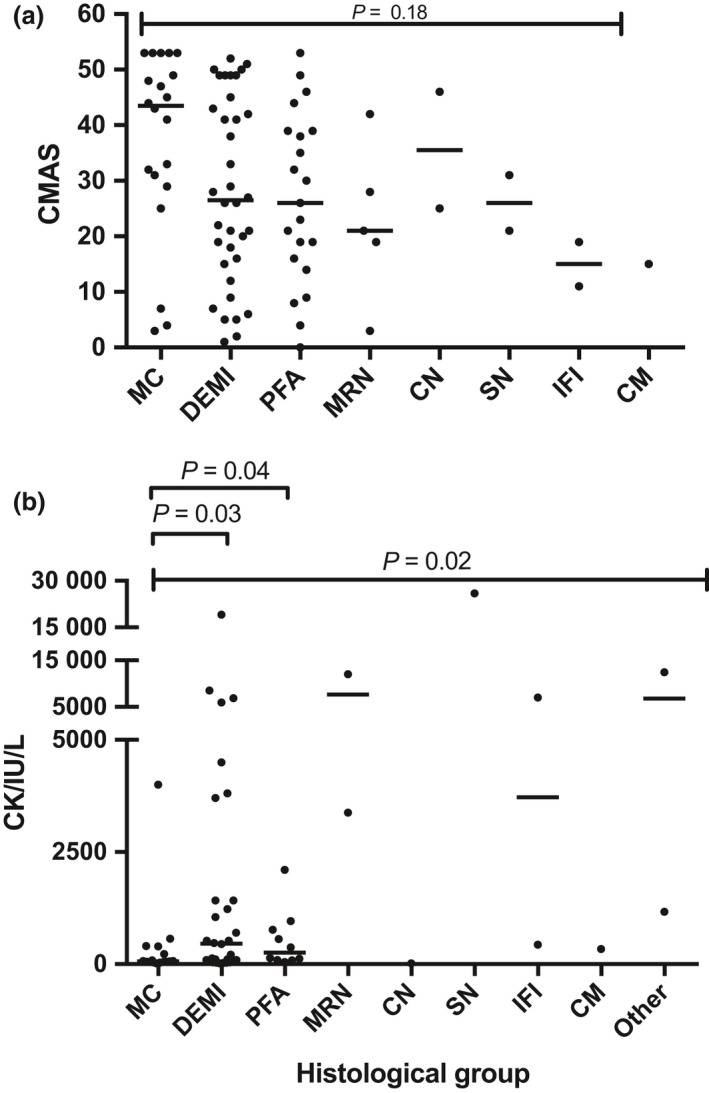
Correlation of (**a**) CMAS score and (**b**) CK levels with histological groups. CMAS scores were available for 88 untreated patients while CK levels were available for 73 untreated patients. Groups with small sample size (*n* < 2) were excluded from the statistical analysis. Bars indicate median; multiple comparisons by Kruskal–Wallis test, individual comparisons by Mann–Whitney test. CMAS, Childhood Myositis Assessment Scale; MC, minimal change; DEMI, diffuse endomysial macrophage infiltrates; PFA, perifascicular atrophy; MRN, macrophage rich necrosis; CN, clustered necrosis; SN, scattered necrosis; IFI, inflammatory fibre invasion; CM, chronic myopathic change.

Juvenile dermatomyositis (probable or definite by Bohan and Peter criteria; 24, 25) was by far the most frequent clinical diagnosis in our cohort (88%) (Table 1). Clinical diagnoses are listed with corresponding histological patterns in Table 4. Given the small numbers of patients with clinical diagnoses other than juvenile dermatomyositis, it is not possible to draw definite conclusions from these data. No statistically significant correlation of histological patterns and CMAS scores, which were available for 88/101 patients, emerged (Figure 6 **a**). Although the MC group shows an aggregate of high CMAS scores, and this is consistent with previous observations in MDA5 +ve JDM, there were also several patients with a low CMAS score in this group, possibly related to the serological heterogeneity of this group as presented in Figure 4.

**Table 4:** Clinical diagnosis and histological groups in 89/101 patients without steroid treatment at the time of diagnosis

	MC	DEMI	PFA	MRN	CN	SN	IFI	CM	Other
JDM	18/89	33/89	17/89	4/89	2/89	1/89	0	1/89	1/89
JDM with scleroderma	1/89	0	1/89	2/89	0	0	2/89	0	0
JDM with chronic polyarthritis	0	1/89	1/89	0	0	0	0	0	0
PM	0	0	1/89	0	0	1/89	0	0	0
Focal myositis	0	0	0	0	0	0	0	0	1/89
Other	0	1/89	0	0	0	0	0	0	0

JDM, juvenile dermatomyositis; PM, Polymyositis; ‘Other’, Inflammatory myopathy clinically not otherwise specified; MC, minimal change; DEMI, diffuse endomysial macrophage infiltrates; PFA, perifascicular atrophy; MRN, macrophage rich necrosis; CN, clustered necrosis; SN, scattered necrosis; IFI, inflammatory fibre invasion; CM, chronic myopathic.

### CK levels show differences across histological patterns

CK levels were available for 73/101 cases (Figure 6 **b**). Only cases without prior steroid treatment were taken into consideration. Upon Kruskal–Wallis testing, there were significant differences between the major histological groups (Figure 6 **b**). The MC histological group showed low CK levels (highest 550 IU/l; Figure 6 **b**;* P *= 0.03 *vs*. DEMI and MRN and *P *= 0.04 *vs*. PFA, Mann–Whitney test) with exception of a single case with a level of 4005 IU/l. The latter case was in the TIF1γ serological group and showed capillary complement deposition, in contrast with the MDA5 cases in the MC group. The wide range of CK levels in the histological group with DEMI infiltrates is remarkable and may suggest that these pathological features on biopsy can indeed be associated with considerable muscle abnormality and, as indicated by the CMAS score, with muscle weakness. There was no statistically significant difference between the PFA and DEMI group (*P *= 0.79, Mann–Whitney test).

## Discussion

This study examines muscle biopsies from a cohort of 101 patients with juvenile immune mediated myopathies to probe potential associations of myopathological features with autoantibody groups. Inclusion in the cohort was based on a clinical diagnosis of juvenile IIM. 90/101 cases underwent serological analysis by immunoprecipitation for myositis‐specific and myositis associated autoantibodies. Seventy percent of cases tested had identifiable autoantibodies. Since biopsy pathology was thus studied in an unbiased cohort with selection criteria which were independent of biopsy results, this study provided an opportunity for a comprehensive analysis of muscle biopsy pathology in this disease group.

Mi2 patients demonstrated consistently high JDM severity scores, displayed a variety of histological patterns including PFA, macrophage rich necrosis and chronic myopathic change, and a tendency towards sarcolemmal rather than capillary complement deposition. The NXP2, TIF1γ and no identifiable autoantibody groups were characterized by a considerable variability in severity scores, a significant proportion of biopsies with DEMI infiltrates and MHC upregulation, several biopsies with PFA, and overall a large proportion of cases with capillary complement deposition. No myopathological differences between these three latter groups were apparent on the present analysis. The increased propensity for sarcolemmal complement deposition in the Mi2‐associated myopathies compared to NXP2 or TIF1γ has been previously reported in adult patients and our data suggest a similar relationship in juvenile IIMs 9. The absence of differences between the NXP2 and TIF1γ on the present morphological analysis and C5b‐9 immunohistochemical profile does not exclude more subtle differences that might exist for instance on a protein expression level or in regard to subgroups of inflammatory cells. Further studies may clarify such potential differences.

Capillary deposition of C5b‐9 was present in a variable proportion in several autoantibody groups in our paediatric cohort. Results from adult TIF1γ‐positive patients indicate that in this age group, C5b‐9 deposition is significantly associated with paraneoplastic myositis 8. Paraneoplastic myositis is rare in children and has not been seen to date in this cohort of 101 cases. The question of whether capillary complement deposition is a biomarker of other disease characteristics in juvenile inflammatory myopathies calls for further study and will be the focus of long‐term outcome and follow up studies in this cohort.

MDA5‐associated myopathies generally had mild pathology according to the JDM score‐tool, did not show DEMI infiltrates or other inflammatory infiltrates, and lacked capillary C5b‐9 deposition. These cases cluster in the histological ‘MC’ group. The absence of major morphological changes in biopsies from juvenile MDA5 patients is in keeping with previous reports of minimal fibre pathology in both juvenile and adult cohorts of anti‐MDA5 associated dermatomyositis 6, 13, suggesting that paediatric MDA5 cases demonstrate similar myopathology to adult cases. It remains to be seen whether changes in nitric oxide synthase expression, as shown in biopsies from adult patients, could be replicated in a paediatric cohort.

Our data suggest a pathomechanistic role for diffuse macrophage infiltrates, which are characteristic of the DEMI group. DEMI histology was found in a large proportion of patients from several serological groups and clinically often typical dermatomyositis. It has a serological profile distinct from the MC group, a different distribution of CK values, and different complement deposition patterns. Endomysial macrophages secrete the highly pro‐inflammatory protein MRP8/14 in juvenile dermatomyositis 26, supporting a significant pathophysiological role for this cell population. Further studies on the subtypes and pathogenetic contribution made by these tissue macrophages are warranted.

The rare myopathological manifestations of MSA‐associated inflammatory myopathies are an interesting group in our cohort. Previous publications on these are available mostly as small series of adult patients with pathologies such as macrophage rich inflammation and clusters of necrotic fibres 17, 18. Similar patterns are present in this paediatric cohort, in small numbers in several serological groups. CN was previously reported as a characteristic of regional ischemic immune myopathy and was suspected to be associated with NXP2 autoantibodies in the original series 18. Our data suggest a similar association in juvenile IIMs. Their potential clinical significance in paediatric patients is largely unknown and deserves further study.

MAA‐associated biopsies show a spectrum of pathologies often similar to those that have been reported in adult cases. Invasion of healthy appearing fibres by inflammatory cells is a feature associated with traditional myopathological descriptions of polymyositis and has been reported in association with PmScl antibodies in adult patients 23. Scattered necrosis is typically seen in immune mediated necrotizing myopathies 10. In our cohort, this occurred exclusively in the SRP group suggesting that immune mediated necrotizing myopathy associated with SRP has similar muscle pathology in adults and children. We have previously reported these cases 14.

The international JDM score tool has been systematically standardized and its application across this large cohort produced robust evidence of a correlation between low scores in the MDA5 and high scores in the Mi2 serological groups, while the NXP2 and TIF1γ positive groups of patients showed a wide range of scores. Scoring also produced an array of data from different histopathological domains, which is amenable to advanced cluster analysis. This type of analysis appears well‐suited to muscle biopsies given that histopathological assessments normally integrates numerous morphological findings. PCA revealed clusters defined by inflammatory and fibre pathology parameters, indicating a high discriminatory value of these domains. Interestingly, assessment of these domains shows the highest interobserver agreement on validation studies 15. The present study combines quantitative evaluation and histopathological classification to create a robust overview of myopathological changes in a large cohort.

The descriptive histological categories used in this study correlate poorly to not at all with clinical diagnosis and CMAS score. There are likely multiple factors causing this variability. The pathophysiology of muscle weakness in inflammatory myopathies is poorly understood and by implication their correlation with morphological change is not obvious. CK levels correlate somewhat better with histological groups than CMAS values probably for similar reasons given that the former reflects muscle fibre degeneration and loss of membrane integrity.

A challenge for any study of pathological subgroups of IIM is to analyse the great variety of myopathological findings that can be seen in order to allow correlation with clinical or serological classifications. The JDM score tool delivers a standardized assignment of a severity score but was not designed to convey all aspects of individual myopathological change. To complement the score tool data, we reviewed all biopsies and chose to group them in descriptive categories based on the dominant pathological features seen in each biopsy. The histological categories used are modelled on major pathologies previously described in the myopathological literature on inflammatory myopathies. In some cases, precise definitions are difficult since the visual interpretation of ‘significant atrophy’, for instance, may vary from one pathologist to another. Yet, these categories are meant to uncover basic patterns and not to serve as discriminators for diagnostic entities. The fact that no apparent differences were disclosed between the NXP2, TIF1γ group and the group without identifiable autoantibodies does not exclude possible differences between these groups in regard to other markers of pathology. Importantly, this study did not aim to define diagnostic criteria using histological features. The strength of our study resides in an unbiased overview of a large cohort with a diverse autoantibody spectrum.

In conclusion, we have mapped myopathological manifestations in a large cohort of patients with clinically and serologically defined juvenile IIM using a dual approach with evaluation based on the validated JDM score tool in a first stage and histological review and grouping in a second. Our results show certain biopsy characteristics of the MDA5 and Mi2 serological groups, but no obvious differences between NXP2, TIF1γ and the group with NIA. Endomysial macrophage infiltrates were a prominent feature in many biopsies with the notable exception of the MDA5 group, supporting a pathomechanistic role for tissue macrophages. Biopsies from myositis‐specific autoantibody groups demonstrated histological variability and variable proportions of capillary C5b‐9 deposition. The clinical and pathomechanistic significance of this variability deserves further study.

## Funding

Funding for the UK JDM Cohort and Biomarker study has been provided by generous grants from the Wellcome Trust UK (085860), Action Medical Research UK (SP4252), The Myositis Support Group UK, Arthritis Research UK (14518, 20164), The Henry Smith Charity and Great Ormond Street Children's Charity (V1268), and the National Institute for Health Research (NIHR) Translational Research Collaboration (TRC) Rare Diseases. This research was supported by the NIHR Biomedical Research Centre at Great Ormond Street Hospital for Children NHS Foundation Trust and GOS Institute of Child Health University College London (UCL). The JDM Cohort study is adopted onto the NIHR Comprehensive Research Network. The Arthritis Research UK Centre for Adolescent Rheumatology at UCL, UCL Hospital and GOSH is supported by grants from Arthritis Research UK [20164] and Great Ormond Street Children's Charity. This is a summary of independent research funded by the NIHR's Rare Diseases Translational Research Collaboration and NIHR Biomedical Research Centre at Great Ormond Street Hospital; LW and CD were supported by the NIHR Biomedical Research Centre at Great Ormond Street Hospital. The views expressed are those of the authors and not necessarily those of the NHS, the NIHR or the Department of Health.

## Author contributions

SAY: JDM score tool analysis, interpretation of data, drafting and revision of manuscript; PWS: Histological analysis, interpretation of data, drafting and revision of manuscript; CTP: Statistical analysis and interpretation, PCA; ES: Score tool evaluation; HV: Immunohistochemical procedures and biobanking; SS: Database administration and analysis; SLT and NJM: Autoantibody analyses and interpretation; JLH: Study design, interpretation of data and revision of manuscript; TSJ: Study design, interpretation of data and revision of manuscript; LRW: Study design, interpretation of data, funding, manuscript revision.

## Conflict of interest

The authors declare no conflict of interest. The Editors of *Neuropathology and Applied Neurobiology* are committed to peer‐review integrity and upholding the highest standards of review. As such, this article was peer‐reviewed by independent, anonymous expert referees and the authors (TJ and JH) had no role in either the editorial decision or the handling of the paper.
